# Mutation status of the KMT2 family associated with immune checkpoint inhibitors (ICIs) therapy and implicating diverse tumor microenvironments

**DOI:** 10.1186/s12943-023-01930-8

**Published:** 2024-01-15

**Authors:** Dong-Xu Wang, Jun-Yu Long, Rui-Zhe Li, Dao-Lin Zhang, Hui Liu, Jingru Liu, Jin-Cheng Tian, Han Li, Jie Liu, Hai-Tao Zhao, Tao Li

**Affiliations:** 1 Department of General Surgery, Qilu Hospital, Shandong University, 107 West Wen Hua Road, Jinan, Shandong 250012 People’s Republic of China; 2 Department of Liver Surgery, State Key Laboratory of Complex Severe and Rare Diseases, Peking Union Medical College Hospital, Chinese Academy of Medical Sciences and Peking Union Medical College (CAMS & PUMC), Beijing, China; 3 Department of Radiation Oncology, Shandong Provincial Key Laboratory of Radiation Oncology, Shandong Cancer Hospital and Institute, Shandong First Medical University, Shandong Academy of Medical Sciences, Shandong University Cancer Center, Jinan, Shandong China

**Keywords:** ICI therapy, KMT2 gene family, Tumor microenvironment, Pan-cancer analysis

## Abstract

Mounting evidence suggests a strong association between tumor immunity and epigenetic regulation. The histone-lysine N-methyltransferase 2 (KMT2) family plays a crucial role in the methylation of histone H3 at lysine 4. By influencing chromatin structure and DNA accessibility, this modification serves as a key regulator of tumor progression and immune tolerance across various tumors. These findings highlight the potential significance of the KMT2 family in determining response to immune checkpoint inhibitor (ICI) therapy, which warrants further exploration. In this study, we integrated four ICI-treated cohorts (*n* = 2069) across 10 cancer types and The Cancer Genome Atlas pan-cancer cohort and conducted a comprehensive clinical and bioinformatic analysis. Our study indicated that patients with KMT2 family gene mutations benefited more from ICI therapy in terms of overall survival (*P* < 0.001, hazard ratio [HR] = 0.733 [95% confidence interval (CI): 0.632–0.850]), progression-free survival (*P* = 0.002, HR = 0.669 [95% CI: 0.518–0.864]), durable clinical benefit (*P* < 0.001, 54.1% vs. 32.6%), and objective response rate (*P* < 0.001, 40.6% vs. 22.0%). Through a comprehensive analysis of the tumor microenvironment across different KMT2 mutation statuses, we observed that tumors harboring the KMT2 mutation exhibited enhanced immunogenicity, increased infiltration of immune cells, and higher levels of immune cell cytotoxicity, suggesting a propensity towards a “hot tumor” phenotype. Therefore, our study indicates a potential association between KMT2 mutations and a more favorable response to ICI therapy and implicates different tumor microenvironments associated with ICI therapy response.

**Supplementary Information:**

The online version contains supplementary material available at 10.1186/s12943-023-01930-8.

## Background

Histone methylation is a common DNA modification that plays a critical role in the regulation of gene transcription. This modification predominantly occurs on lysine residues at the N-terminus of histones and is catalyzed by a family of enzymes known as histone-lysine N-methyltransferases (KMT) [1]. Members of the histone-lysine N-methyltransferase 2 (KMT2) family comprise several subtypes, including KMT2A, KMT2B, KMT2C, and KMT2D, which exert significant effects on cellular processes such as proliferation, growth, development, and differentiation at various stages.

Emerging research has demonstrated that mutations or aberrant expression of KMT2 genes are frequently observed in various tumors. These alterations disrupt histone methylation, leading to dysregulation of DNA damage repair, gene expression, and chromosomal structure, ultimately affecting their normal functions [2]. Consequently, these abnormalities directly contribute to the accumulation of genomic instability, increasing the risk of genetic mutations and chromosomal aberrations, thereby promoting tumor initiation and progression. Furthermore, genomic instability in tumor cells enhances tumor immunogenicity, potentially improving sensitivity to immune checkpoint inhibitor (ICI) therapy [3]. As a result, there is growing interest in investigating the role of KMT2 genes in tumor immunotherapy. In colorectal cancer, KMT2 family mutations are associated with a higher tumor mutation burden (TMB) and microsatellite instability, which are correlated with improved prognosis in patients with colorectal cancer [1, 4]. In non-small cell lung cancer (NSCLC), different genetic alterations are associated with varying levels of programmed death-ligand 1 expression and TMB [5]. The co-occurrence of TP53/KMT2C mutations can effectively predict the response to ICI treatment. Collectively, these findings highlight the importance of understanding the impact of KMT2 genes on tumor immunotherapy, offering potential avenues for targeted therapies and personalized treatment strategies.

However, current research has primarily focused on individual genes within the KMT2 family or specific cancer types, lacking comprehensive investigations into the systemic effects of the entire KMT2 family and their impact on the tumor immune microenvironment [6]. Therefore, it is crucial to conduct a systematic analysis using multiple immunotherapy cohorts and pan-cancer databases that can provide extensive genetic profiling characteristics [7–10]. This study aimed to explore the response to ICI therapy and the intrinsic biological connections in KMT2-mutated tumors across various dimensions, including immunotherapeutic efficacy and the tumor immune microenvironment. Through examining multiple characteristics, this study aimed to provide robust evidence regarding the relationship between KMT2 alterations and tumor immunotherapy, potentially contributing to advancements in this field.

## Methods

This study integrated mutational and clinical information from ICI-treated patients across four studies. MSK-IMPACT and whole-exome sequencing (WES) were utilized to sequence cohort samples, classifying tumors as KMT2-MUT or KMT2-WT based on KMT2 non-synonymous somatic mutations. Data from The Cancer Genome Atlas (TCGA) pan-cancer cohort across 33 cancer types were obtained to study the prognostic impact of KMT2 family mutations and the different tumor microenvironments between KMT2-WT and KMT2-MUT tumors.

Outcome measures, including the objective response rate (ORR), overall survival (OS), progression-free survival (PFS), and durable clinical benefit (DCB), were obtained from four studies. The TMB was calculated differently for the MSK-IMPACT and WES-sequenced samples. The proportion of infiltrating immune cells was determined using the CIBERSORT algorithm. Immune cell scores from a pan-cancer study and the geometric means of granzyme A and perforin 1 for the cytolytic activity score (CYT) were calculated. Immunogenomic indicators, 29 immune signatures and 10 oncogenic pathways enrichment scores were obtained from various sources. Statistical analyses involved Fisher’s exact test, log-rank test, Cox regression analysis, and Wilcox test using R software (version 4.0.2; Foundation for Statistical Computing, Vienna, Austria). Statistical significance was set at *P* < 0.05. For detailed content and references, please refer to the Supplementary Methods.

## Results

### Mutation status of the KMT2 family in the TCGA pan-cancer cohort

**Fig. 1:**
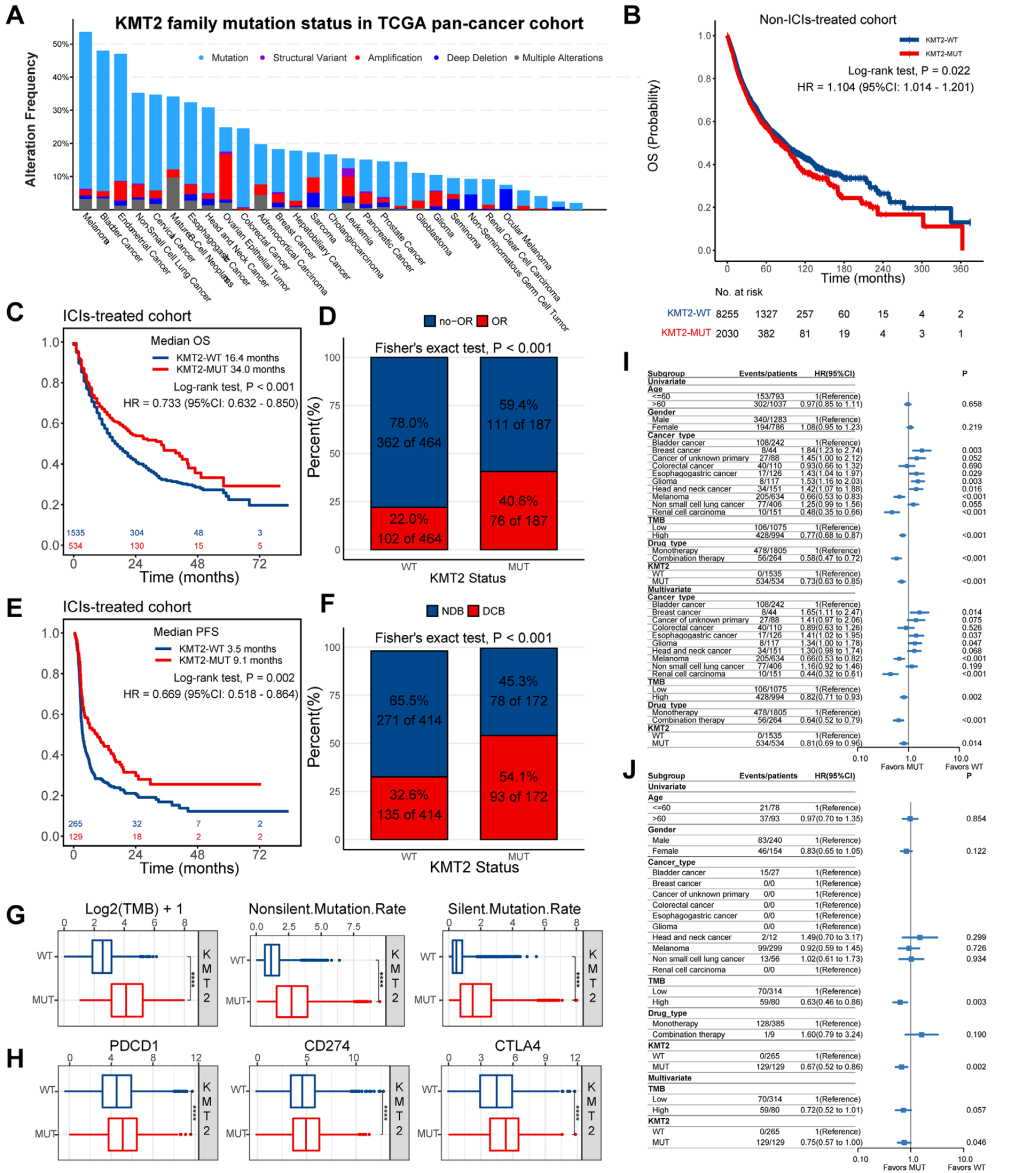
KMT2 family mutations predicted better clinical outcomes in the ICI-treated cohort. **A** Scale stacked column chart of the mutation status of the KMT2 family in the TCGA pan-cancer cohort. **B** K-M curve shows the difference of OS between patients with and without KMT2 family mutations in the TCGA pan-cancer cohort. **C** K-M curves show the difference of OS between patients with and without KMT2 family mutations in the ICI-treated cohort (*n* = 2069). **D** Patients with KMT2 family mutations exhibited a high ORR while receiving ICI therapy. **E** K-M curves show the difference of PFS between patients with and without KMT2 family mutations in the ICI-treated cohort. **F** Patients with KMT2 family mutations exhibited high ORRs and DCBs while receiving ICI therapy. **G** The TMB levels for patients with and without KMT2 family mutations in the ICI-treated cohort (*n* = 2069), Non-silent and silent mutation rates of patients with and without KMT2 family mutations in the TCGA pan-cancer cohort. **H** The mRNA expression levels of three immune checkpoints (PDCD1, CD274, CTLA-4) for patients with and without KMT2 family mutations in the TCGA pan-cancer cohort. **I, J** OS- or PFS-related univariate and multivariate Cox regression analyses revealed that KMT2 family mutations are a potential independent predictor for the prognosis of patients receiving ICI therapy

This study explored the somatic alteration frequency of four KMT2 genes (KMT2A, KMT2B, KMT2C, and KMT2D) in the TCGA pan-cancer cohort. The analysis revealed a high mutation rate within the KMT2 family across various cancers, with melanoma exhibiting the highest rate (exceeding 50%, Fig. 1A). In the TCGA pan-cancer cohort, the mutational landscape of the KMT2 gene family revealed that KMT2D and KMT2C exhibited the highest mutation frequency at 10%, followed by KMT2A and KMT2B at 6% (Fig S1A). However, no significant hotspot mutations were identified in the KMT2 family (Fig S1B). Survival analysis demonstrated that patients with KMT2 family mutations had decreased OS (Fig. 1B), whereas no significant survival difference was observed in patients with or without mutations of the individual members of the KMT2 family (Fig S2). Considering the influence of clinical and pathological factors, we conducted subgroup analyses based on the tumor type, clinical stage, and histological grade. Univariate Cox regression analyses revealed that KMT2 family mutations significantly affected the prognosis of patients in the malignant pleural mesothelioma and uterine corpus endometrial carcinoma subgroups (Table S1). No significant differences were observed among the other subgroups (Table S1-3).

### KMT2 family mutations predicted improved clinical outcomes in the ICI-treated cohort

We constructed an ICI-treated cohort comprising response data and mutational data from four studies (*n* = 2069) across 10 cancer types to explore the impact of KMT2 family mutations on clinical outcomes in patients receiving ICI therapy. Patients were divided into the KMT2 (A, B, C, D)-MUT and KMT2-WT groups according to the family or individual KMT2 mutation status. First, we explored the difference in TMB between the KMT2-MUT and KMT2-WT groups in the ICI-treated cohort and found that the KMT2-MUT group had a higher TMB (Fig. 1G); similar results were observed in the KMT2 (A, B, C, D)-MUT group (Fig S1C). In the TCGA pan-cancer cohort, we found that non-silent and silent mutation rates were higher in the KMT2-MUT (Fig. 1G) and KMT2 (A, B, C, D)-MUT groups compared with the KMT2-WT group (Fig S1D, E). These results suggested that KMT2-MUT tumors had improved immunogenicity. The mRNA expression levels of three immune checkpoints (PDCD1, CTLA-4, CD274) were compared between the KMT2-MUT and KMT2-WT group, and they were all significantly elevated in the KMT2-MUT group (Fig. 1H). Similar results were observed in the KMT2 (A, B, C, D)-MUT group (Fig S3A), suggesting that KMT2-MUT tumors might be sensitive to ICI therapy. Moreover, we analyzed clinical outcomes (PFS, OS, DCB, and ORR) in the KMT2-MUT and KMT2-WT groups. The results indicated that the KMT2-MUT group had significantly longer OS (median OS: 34.0 months vs. 16.4 months, *P* < 0.001, hazard ratio [HR] = 0.733 [95% confidence interval (CI): 0.632–0.850]) and PFS (median PFS: 9.1 months vs. 3.5 months, *P* = 0.002, HR = 0.669 [95% CI: 0.518–0.864]) (Fig. 1C, E), and significantly higher ORR (40.6% vs. 22.0% *P* < 0.001) and DCB (54.1% vs. 32.6% *P* < 0.001) (Fig. 1D, F). Similar results were observed in the KMT2(A, B, C, D)-MUT group (Fig S3B-E). OS- or PFS-related univariate and multivariate Cox regression analyses were further conducted, and we found that mutations in the KMT2 family are potential independent predictor for the prognosis of patients receiving ICI therapy (Fig. 1-I, J); similar results were observed in the KMT2 (A, B, C, D)-MUT group (Fig S4). Using random sampling for 1000 iterations, we analyzed the clinical outcomes (PFS, OS, DCB, and ORR) within each generated internal clinical cohort. Patients in the KMT2-MUT group exhibited longer average median OS (34.00 vs. 16.58 months, HR = 0.574 [95% CI: 0.617–0.877]) and PFS (9.07 vs. 3.55 months, HR = 0.667 [95% CI: 0.498–0.894]), along with higher average ORR (40.6% vs. 22.0%) and DCB (54.4% vs. 33.3%) (Supplementary file 3).

### Exploration of tumor immune microenvironment in KMT2-MUT and KMT2-WT tumors

**Fig. 2:**
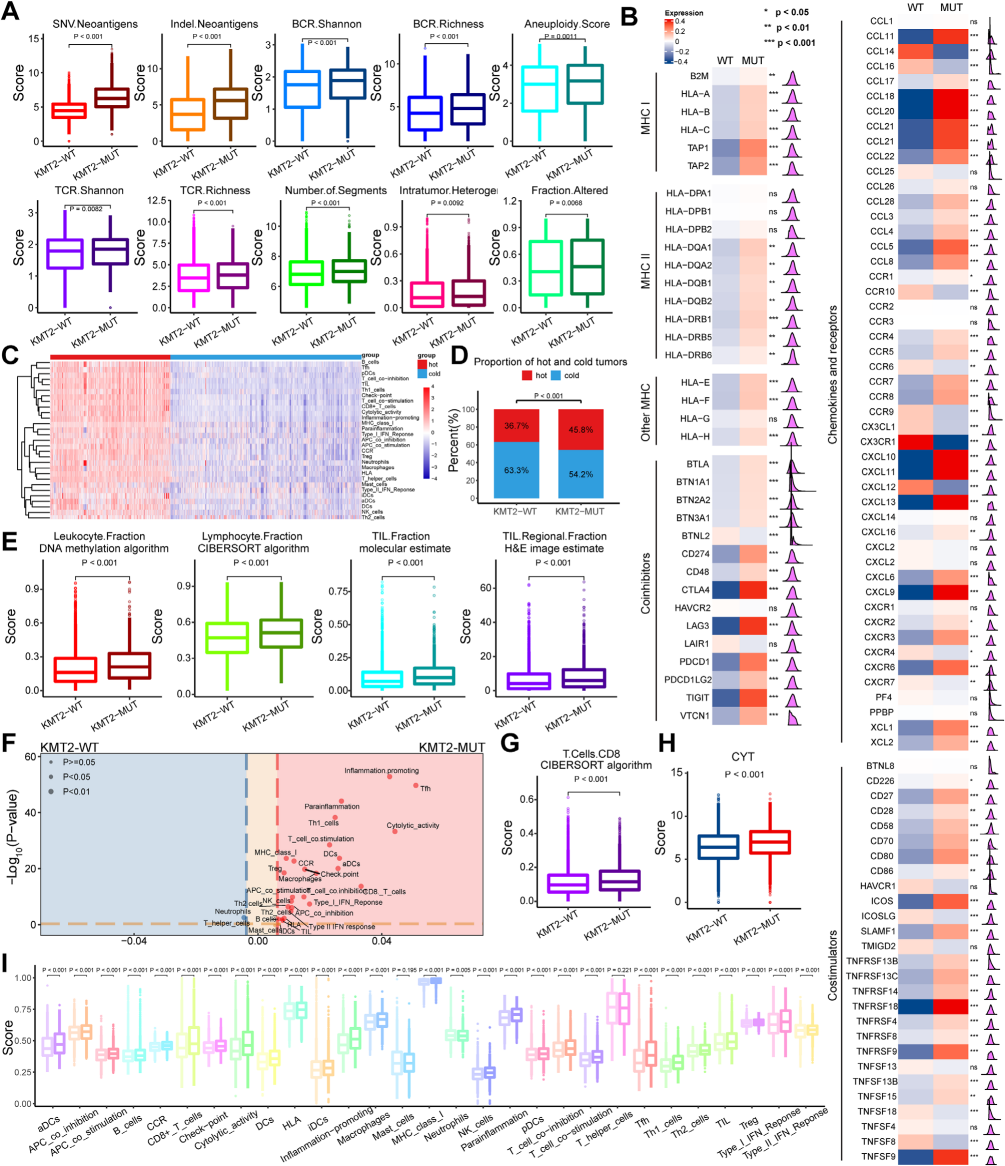
Exploration of the tumor microenvironment in KMT2-MUT and KMT2-WT tumors. **A** Comparison of the scores for 10 immunogenomic indicators between KMT2-MUT and KMT2-WT tumors. **B** The difference of mRNA expression levels in MHC molecules, coinhibitors, chemokines, receptors, and costimulators between KMT2-MUT and KMT2-WT tumors. **C** Two stable immune subtypes defined as “hot tumor” and “cold tumor” were identified using an unsupervised cluster. **D** A higher proportion of “hot tumors” was observed in tumors with KMT2 family mutations. **E** Differences in leukocyte fraction (DNA methylation algorithm), lymphocyte fraction (CIBERSORT algorithm), TIL fraction (molecular estimate), and the TIL regional fraction (H&E image estimate) between KMT2-MUT and KMT2-WT tumors. **F** The volcanic diagram provides a more in-depth presentation of higher immune signature scores in KMT2-MUT tumors. **G** Comparison of CD8 T cells fraction (CIBERSORT algorithm) between KMT2-MUT and KMT2-WT tumors. **H** Comparison of CYT between KMT2-WT and KMT2-MUT tumors. **I** Comparison of 29 immune signature scores estimated using the “ssGSEA” method between KMT2-MUT and KMT2-WT tumors

Tumor immunogenicity plays a critical role in antitumor immunity, and boosted tumor immunogenicity stimulates improved antitumor immunity. We compared the scores of 10 immunogenomic indicators between KMT2-MUT and KMT2-WT tumors and found that the scores of all 10 immunogenomic indicators were significantly higher in KMT2-MUT tumors (Fig. 2A). We also observed that the expression levels of most MHC molecules were elevated in KMT2-MUT tumors (Fig. 2B). These results suggest that KMT2-MUT tumors have significantly boosted immunogenicity.

Immune cell infiltration into tumors is crucial for the immune system to execute immune functions. We compared the immune cell infiltration levels between KMT2-MUT and KMT2-WT tumors from four aspects: (1) leukocyte fractions were measured using DNA methylation arrays; (2) infiltration levels of lymphocytes, measured using the CIBERSORT algorithm; (3) genomic measurements of the tumor-infiltrating lymphocyte (TIL) fraction; and (4) the TIL fraction estimated by deep learning methods based on hematoxylin and eosin-stained (H&E-stained) slides. The results demonstrated that the scores of all four indicator were higher in KMT2-MUT tumors (Fig. 2E) compared with the KMT2-WT tumors, suggesting that KMT2-MUT tumors exhibited higher levels of immune cell infiltration. Similar results were observed in KMT2 (A, B, C, D)-MUT tumors (Fig S5). Twenty-nine immune signature scores of each sample in the TCGA pan-cancer cohort were estimated using the “single-sample gene set enrichment analysis (ssGSEA)” method. Based on these immune signature scores, two stable immune subtypes were identified using unsupervised clustering. The immune subtype with higher immune signature scores was defined as “hot tumor” while the immune subtype with lower immune signature scores was defined as “cold tumor” (Fig. 2C). After conducting Fisher’s exact test, we found that a significantly higher proportion of “hot tumors” existed in KMT2-MUT tumors (Fig. 2D); similar results were observed in KMT2 (A, B, C, D)-MUT tumors (Fig S6A). We then used Danaher’s method to estimate the enrichment scores of particular cells in each sample of the TCGA pan-cancer cohort. Most enrichment scores, including those of CD8 T cells, were higher in KMT2-MUT tumors than in KMT2-WT tumors (Fig. S6B). Considering that CD8 T cells are critical for antitumor immunity, we also estimated the CD8 T cell proportion of each sample in the TCGA pan-cancer cohort using the CIBERSORT algorithm and determined that KMT2-MUT tumors (Fig. 2G) had a larger proportion of CD8 T cell compared with KMT2 (A, B, C, D)-MUT tumors (Fig S7). The volcanic diagram provides a more in-depth presentation of the higher cell enrichment scores in KMT2-MUT tumors (Fig S8A).

The expression levels of some chemokines, such as CXCL9 and CXCL10, that have been shown to recruit CD8 T cells, were significantly higher in KMT2-MUT tumors (Fig. 2B). This association might explain the higher immune cell infiltration levels in KMT2-MUT tumors. Twenty-nine immune signature scores possibly representing the immune activity profile of the tumor in each sample of the TCGA pan-cancer cohort were estimated using the “ssGSEA” method. The results indicated that most immune signature scores were higher in KMT2-MUT tumors. The volcanic diagram provides a more in-depth presentation (Fig. 2F, I). Additionally, the scores for CD8 T cells, which play a key role in tumor immunity, were significantly higher in KMT2-MUT tumors. Moreover, the correlation among immune activities was higher in KMT2A-MUT tumors (Fig S8B, C), while no significant difference was observed between the other two groups. (Fig S9A). We also calculated the CYT to evaluate the differences in immune cell cytotoxicity between KMT2-MUT and KMT2-WT tumors and found that KMT2-MUT tumors had stronger immune cell cytotoxicity (Fig. 2H; S8E, F; S9B-D). In addition, the expression levels of most interleukins and receptors were higher in KMT2-MUT tumors (Fig S8G). These results indicated that boosted tumor immunogenicity, higher levels of immune infiltration, and improved immune activity exist in KMT2-MUT tumors, suggesting that KMT2-MUT tumors might have an improved response to ICI therapy. In addition, we investigated some classical carcinogenic pathways enriched in KMT2-MUT and KMT2-WT tumors, the results of which are shown in Fig S8H.

To explore the consistency of our research findings across various immune infiltration assessment methods, we included a comprehensive table summarizing the results obtained from various algorithms, such as XCELL, EPIC, MCPCounter, and ESTIMATE (Supplementary File 1). We employed the ESTIMATE algorithm to assess all samples in the TCGA pan-cancer cohort and discovered that KMT2-MUT tumors exhibit a significantly higher “ImmuneScore,” indicating a greater degree of overall immune infiltration in KMT2-MUT tumors.

## Discussion

Our study results demonstrated that KMT2 family mutations could predict improved clinical outcomes in patients undergoing ICI therapy. This could be attributed to the enhanced tumor immunogenicity, increased immune infiltration, and improved immune activity observed in KMT2-MUT tumors. The tumor microenvironment characterized by these factors may play a crucial role in facilitating better clinical outcomes in patients with KMT2 family mutations receiving ICI therapy.

As an important epigenetic regulator, KMT2 frequently undergoes frameshift, truncation, and missense mutations in various tumors, mainly affecting the expression of the carboxyl-terminal SET domain. KMT2C is the most commonly mutated gene in gastric adenocarcinoma, while KMT2D is one of the most frequently mutated genes in epithelial cancers. Epithelial tissues rely on a highly coordinated balance among self-renewal, proliferation, and differentiation. KMT2D mutations can disrupt this balance and drive the transformation of the normal epithelium into tumors. This is because KMT2D mutations can lead to decreased expression of p63 target genes and key genes involved in epithelial development and adhesion as well as widespread loss of histone enhancer modifications H3K4 monomethylation and H3K27 acetylation [11].

In our study, four KMT2 family genes (KMT2A, KMT2B, KMT2C, and KMT2D) were identified as the most commonly altered genes in various tumors. To ensure that the OS difference between patients with different KMT2 family statuses treated with ICIs was not solely attributable to the general prognostic benefits of KMT2 family mutations, we conducted a survival analysis. This analysis compared patients with or without KMT2 family mutations in the TCGA pan-cancer cohort, aiming to investigate the general prognostic impact of KMT2 family mutations in various cancer types. Our results indicated a trend towards poorer prognosis among untreated patients with KMT2 mutations; however, no statistical significance was achieved. Subsequently, within specific ICI therapy cohorts, we found that, after initiating ICI therapy, patients with mutations demonstrated significantly better survival than those without mutations. This suggests that mutations in KMT2 may lead to biological changes in the tumor, consequently rendering these cancers more responsive to ICI treatment. Zhang et al. found that patients with KMT2A/C mutations had improved prognosis in terms of PFS, ORR, DCB, and OS [12]. These findings align with those of our research; however, their study did not systematically categorize the KMT2 family genes or investigate the tumor microenvironment to explore their potential mechanisms. Research exploring the relationship between KMT2 and immunotherapy is lacking. To verify the reliability of our results, we randomly selected a number of patients from the KMT2-WT group similar to that of the KMT2-MUT group, forming an internal clinical cohort along with patients from the KMT2-MUT group. These results confirmed the conclusions obtained from the original ICI-treated cohort.

Studies on the structure of the KMT2 gene and its corresponding proteins are gradually increasing, clarifying its potential as a target drug [2]. However, research on changes in the tumor microenvironment caused by KMT2 gene alterations and its specific role in tumor immune regulation is scarce [13]. In this study, we explored the tumor microenvironment of KMT2-MUT and KMT2-WT tumors in pan-cancer and ICI cohorts. We explored immune infiltration from four aspects, including the leukocyte fraction measured by DNA methylation arrays, the infiltration levels of lymphocytes measured by the CIBERSORT algorithm, genomic measurement of the TIL fraction, and the TIL fraction estimated by deep learning methods based on H&E-stained slides [14, 15]. In addition, we have included a comprehensive table summarizing the results obtained from various algorithms, such as XCELL, EPIC, MCPCounter, and ESTIMATE. These results indicate a greater degree of immune infiltration in KMT2-MUT tumors compared with KMT2-WT tumors. However, the function, molecular mechanism, and biological significance of KMT2 mutations in tumors require further study. This will potentially aid in discovering improved targeted treatment methods and positively impact personalized cancer treatment.

Our current research has some limitations and lacks experimental studies. Therefore, further clinical and fundamental experiments are needed to explore the mechanisms underlying KMT2-MUT tumors. Compared to combined prognostic signatures, single-gene family analysis does not provide a more comprehensive and detailed picture of the biological and/or clinical significance of the findings.

## Conclusion

This study revealed that patients with KMT2 mutations benefited significantly from ICI therapy in terms of OS, PFS, DCB, and ORR. These tumors are considered “hot tumors,” harboring a tumor microenvironment potentially more responsive to ICI therapy. Consequently, KMT2 family mutation status could serve as an effective predictor of ICI therapy outcomes, indicating associated tumor microenvironment variations and guiding personalized therapeutic strategies.

### Electronic supplementary material

Below is the link to the electronic supplementary material.

Supplementary Material 1

Supplementary Material 2

Supplementary Material 3

Supplementary Material 4

## Data Availability

All of the data we used in this study were publicly available in cBioPortal (https://www.cbioportal.org) and the PanCancer Atlas consortium (https://gdc.cancer.gov/about-data/publications/pancanatlas). The code utilized for data processing to support the findings of this study is available on request from the corresponding author.

## Abbreviations

CNN: Convolutional neural network
CR: Complete response
CTLA-4: Cytotoxic T lymphocyte antigen 4
CYT: Cytolytic activity score
DC: Dendritic cell
DCB: Durable clinical benefit
FDA: Food and Drug Administration
GZMA: Granzyme A
H&E-stained: Hematoxylin and eosin-stained
HNSCC: Head and neck squamous cell carcinoma
HR: Hazard ratio
IPS: Immunophenoscore
ICIs: Immune checkpoint inhibitors
KMT2: Histone-lysine N-methyltransferase 2
KMT2-WT: KMT2-wildtype
KMT2-MUT: KMT2-mutant
MHC: Major histocompatibility complex
MSK-IMPACT: Memorial Sloan Kettering-Integrated Mutation Profiling of Actionable Cancer Targets
NDB: No durable benefit
NSCLC: Non-small cell lung cancer
ORR: Objective response rate
OS: Overall survival
PD: Progression of disease
PD-(L)1: Programmed cell death (ligand) 1
PFS: Progression-free survival
PR: Partial response
PRF1: Perforin 1
RECIST: Response Evaluation Criteria in Solid Tumors
SD: Stable disease
ssGSEA: Single-sample gene set enrichment analysis
TCGA: The Cancer Genome Atlas
TCR: T cell receptor
TIL: Tumor-infiltrating lymphocyte
TMB: Tumor mutational burden
TME: Tumor microenvironment
WES: Whole-exome sequencing
