# Supplementary material for: Mutation status of the KMT2 family associated with immune checkpoint inhibitors (ICIs) therapy and implicating diverse tumor microenvironments
Source: Mol Cancer. 2024 Jan 15;23:15. doi: 10.1186/s12943-023-01930-8 (PMC10789049; doi:10.1186/s12943-023-01930-8)
Supplement: Supplementary file 1 — Supplementary Material 1 [file 12943_2023_1930_MOESM1_ESM.docx]

| HR | Pvalue | 95%CI | Cancer types |
| --- | --- | --- | --- |
| 1.266 | 0.28085 | 0.825-1.944 | GBM |
| 0.908 | 0.64166 | 0.604-1.365 | OV |
| 1.057 | 0.74506 | 0.756-1.477 | LUAD |
| 0.972 | 0.84537 | 0.73-1.294 | LUSC |
| 1.734 | 0.49644 | 0.355-8.468 | PRAD |
| 0.585 | 0.02293 | 0.368-0.928 | UCEC |
| 0.857 | 0.30748 | 0.637-1.153 | BLCA |
| 0 | 0.99914 | 0-Inf | TGCT |
| 1.107 | 0.71286 | 0.644-1.905 | ESCA |
| 1.29 | 0.41044 | 0.703-2.366 | PAAD |
| 0.736 | 0.5213 | 0.288-1.878 | KIRP |
| 1.124 | 0.63243 | 0.696-1.816 | LIHC |
| 1.106 | 0.68889 | 0.675-1.815 | CESC |
| 0.95 | 0.89078 | 0.458-1.97 | SARC |
| 1.516 | 0.06927 | 0.968-2.376 | BRCA |
| 0 | 0.9983 | 0-Inf | THYM |
| 4.633 | 0.00446 | 1.61-13.325 | MESO |
| 1.128 | 0.58978 | 0.729-1.744 | COAD |
| 0.803 | 0.21578 | 0.567-1.137 | STAD |
| 0.998 | 0.98655 | 0.76-1.31 | SKCM |
| 2.779 | 0.08885 | 0.856-9.021 | CHOL |
| 1.561 | 0.12334 | 0.886-2.752 | KIRC |
| 0 | 0.99803 | 0-Inf | THCA |
| 1.123 | 0.42675 | 0.843-1.497 | HNSC |
| 0.423 | 0.14456 | 0.133-1.344 | LAML |
| 0.218 | 0.13714 | 0.029-1.623 | READ |
| 0.745 | 0.52221 | 0.303-1.835 | LGG |
| 0.417 | 0.43365 | 0.047-3.73 | DLBC |
| 1.973 | 0.52273 | 0.245-15.861 | KICH |
| 0.911 | 0.86166 | 0.318-2.605 | UCS |
| 1.868 | 0.14402 | 0.808-4.321 | ACC |
| 0 | 0.99865 | 0-Inf | PCPG |
| 0 | 0.99744 | 0-Inf | UVM |

Table S1 The results of univariate Cox regression analysis in the subgroups based on different tumor types.

| HR | Pvalue | 95%CI | Stage |
| --- | --- | --- | --- |
| 1.104 | 0.5436 | 0.802-1.521 | Stage I |
| 1.052 | 0.79631 | 0.716-1.545 | Stage II |
| 1.064 | 0.66287 | 0.805-1.406 | Stage III |
| 1.279 | 0.15009 | 0.915-1.787 | Stage IV |

Table S2 The results of univariate Cox regression analysis in the subgroups based on the patients' clinical stages.

| HR | Pvalue | 95%CI | Grade |
| --- | --- | --- | --- |
| 1.319 | 0.23757 | 0.833-2.09 | G1 |
| 1.245 | 0.05209 | 0.998-1.554 | G2 |
| 1.042 | 0.66863 | 0.863-1.258 | G3 |
| 1.07 | 0.92003 | 0.284-4.037 | G4 |

Table S3 The results of univariate Cox regression analysis in the subgroups based on the patients' histological grades.
