# Supplementary material for: Mutation status of the KMT2 family associated with immune checkpoint inhibitors (ICIs) therapy and implicating diverse tumor microenvironments
Source: Mol Cancer. 2024 Jan 15;23:15. doi: 10.1186/s12943-023-01930-8 (PMC10789049; doi:10.1186/s12943-023-01930-8)
Supplement: Supplementary file 2 — Supplementary Material 2 [file 12943_2023_1930_MOESM2_ESM.docx]

**Methods and material**

**Study population and data download**

Mutational data and clinical information from patients treated with ICIs therapy were accessed and integrated from four published studies[1-4]. We collected the mutational data from cBioPortal (https://www.cbioportal.org). Samples from the cohort constructed by Samstein et al and were sequenced utilizing the Memorial Sloan Kettering-Integrated Mutation Profling of Actionable Cancer Targets (MSK-IMPACT) panel, which was a RNA sequencing platform that can detect mutations and was authorized by the US FDA, and the other cohorts were sequenced by whole-exome sequencing (WES). In this study, we included all nonsynonymous mutations consisting of translationm start site, splice site, nonstop, nonsense, frameshift, and missense mutations[5]. Tumor samples were defined as *KMT2*-mutant (*KMT2*-MUT) and *KMT2*-wildtype (*KMT2*-WT) based on if there were *KMT2* non-synonymous somatic mutations. Finally, ten cancer types were included in the ICIs-treated cohort and 2069 cases were remained.

Moreover, to investigate Effect on patient prognosis of KMT2 family mutations and the variations in immune response landscapes between *KMT2*-WT and *KMT2*-MUT tumors, we integrated data from The Cancer Genome Atlas (TCGA) cohort (n = 10,143) of 33 cancer types. The standardized, normalized, batch corrected and platform-corrected data matrices and mutation data generated by the PanCancer Atlas consortium, available at the publication page (https://gdc.cancer.gov/about-data/publications/pancanatlas) were used in this study

The tumor mutational burden (TMB) for MSK-IMPACT panel sequenced samples was sourced from the corresponding literature. In the case of WES-sequenced samples, TMB was defined as the total number of nonsynonymous mutations divided by the exome size (with an exome size of 38 Mb used).

The primary clinical outcomes were ORR, OS, PFS, and clinical benefit. The ORR was obtained from the seven studies according to Response Evaluation Criteria in Solid Tumors (RECIST)version 1.1. Clinical benefit was classified as DCB (partial response [PR/]complete response [CR] or stable disease SD that lasted > 6 months) or no durable benefit (NDB, stable disease [SD] that lasted ≤ 6 months or progression of the disease [PD]) [6] The OS of patients in the non-ICI-treated cohort was calculated from the date when the tumor specimen was collected to the date of the latest follow-up or death. The overall survival (OS) of patients in the ICI-treated cohort was calculated from the time of initiation of ICI therapy until either the last follow-up or death.

In the four ICIs-treated cohorts, the study utilized mutation data and clinical data for analysis. The mutation data is treated as a binary variable, indicating the presence or absence of gene mutations. The study merged the four cohorts directly, keeping the shared mutation data and clinical data. The mRNA expression data profile for TCGA Pan-Cancer, named EBPlusPlusAdjustPANCAN_IlluminaHiSeq_RNASeqV2.geneExp.tsv, was downloaded from the PanCanAtlas. The data was quantified using RSEM. The batch effect-normalized mRNA data were then transformed with log2(norm_value+1).

**CIBERSORT algorithm Evaluating immune infiltration**

CIBERSORT is a deconvolution algorithm for identifying cell composition of bulk cancer tissues of mixed cell types based on their gene expression profiles [7]. The CIBERSORT algorithm was employed to calculate the proportions of 22 types of infiltrating immune cells based on normalized gene expression data and a leukocyte gene signature matrix, termed LM22.CIBERSORT immune infiltration proportions of TCGA samples were accessed from the pancancer immune landscape project conducted by Thorsson et al[8].

**Analysis of TIL fraction, lymphocyte fraction and Leukocyte fraction**

In the TCGA pancancer cohort, the levels of TILs from genomics measurement and those of TILs from H&Estained image measurement were accessed by analyzing the data from Saltz et al. and Torsson et al., respectively[8,9]. Saltz et al. presented global mappings of tumor-infiltrating lymphocytes (TILs) for more than 5,000 H&E-stained diagnostic whole-slide images from the TCGA database using deep learning-based lymphocyte classification with convolutional neural networks (CNNs). This approach is considered a benchmark for TIL analysis. Genomics measurement of the TIL fraction was accessed by multiplying an aggregated proportion of the lymphocyte fraction in the immune compartment estimated by CIBERSORT algorithm with the leukocyte fraction derived from DNA methylation. The lymphocyte fraction is an aggregation of CIBERSORT estimates of naïve and memory B cells, follicular helper T cells, naïve, resting and activated memory CD4 T cells, T regulatory cells, CD8 T cells, resting and activated NK cells, plasma cells, and gamma-delta T cells.

**The immune infltration cell scores from Danaher et al.**

The immune infiltration scores were sourced from a previous pancancer study conducted by Danaher et al. [10] in the TCGA database. Each immune cell score was determined using 60 specific marker genes with expression levels capable of classifying 14 immune cell populations, including total tumor-infiltrating lymphocytes (TILs), B cells, dendritic cells (DCs), macrophages, exhausted CD8+ T cells, CD8+ T cells, neutrophils, cytotoxic cells, regulatory T cells (Tregs), natural killer (NK) CD56dim cells, mast cells, NK cells, and T helper 1 (T1) cells. These results exhibited high reproducibility and concordance with those obtained using immunohistochemistry and flow cytometry.

**Multiple deconvolution algorithms for identifying immune distribution**

The calculation results of multiple deconvolution algorithms for each sample in the TCGA pan-cancer cohort were downloaded from the TIMER2 database(http://timer.cistrome.org/). The calculation results of the ESTIMATE algorithm were obtained using the R package "estimate".

**Cytolytic activity score**

Cytolytic activity score(CYT) was the geometric mean of granzyme A (GZMA) and perforin 1 (PRF1) expression[11].

**Calculation of immunogenomic indicators**

Immunogenomic indicators were acquired from the pancancer immune landscape project conducted by Thorsson et al. [8]. In brief, neoantigen prediction by SNVs was performed using OptiType tool v1.2, NetMHCpan v3.0, and PanCancer MC3 Consortium. Neoantigen prediction by indels was performed using VEP v87 (Ensembl Variant Effect Predictor) and the pVAC-Seq v4.0.8 pipeline with NetMHCpan v3.0. TCR diversity scores (Shannon entropy and richness) were inferred from tumor RNA-seq data.

**Immune signature evaluation**

Twenty-nine classical immune signatures were acquired from He et al.[12]. We used the “GSVA” R package (version: 1.34.0) based on the single-sample gene set enrichment analysis (ssGSEA) method to quantify the enrichment levels of the twenty-nine immune signatures in each sample [13].

**Enrichment scores of oncogenic pathways**

Ten oncogenic pathways, comprising a total of 187 oncogenes, were identified in the study conducted by Sanchez-Vega et al.[14].To determine the enrichment scores for each pathway in each sample, we employed the ssGSEA approach and utilized the "GSVA" R package.

**Statistical analysis**

We used Fisher’s exact test to evaluate the enrichment of KMT2 family mutation status with response (ORR and DCB). Additionally, we employed the log-rank test and Cox proportional hazards regression analysis to analyze the difference in progression-free survival (PFS) and overall survival (OS) between *KMT2(A,B,C,D)*-MUT and *KMT2(A,B,C,D)*WT patients. The data for the comparison between two groups are both ordinal and non-normally distributed. So, to compare two groups, the Wilcox test was performed. All statistical analyses were conducted using R software (version 4.0.2), and P values were two-tailed. A P value less than 0.05 was considered statistically significant.

**Reference**

1 Liu, D. *et al.* Integrative molecular and clinical modeling of clinical outcomes to PD1 blockade in patients with metastatic melanoma. *Nat Med* **25**, 1916-1927, doi:10.1038/s41591-019-0654-5 (2019).

2 Miao, D. *et al.* Genomic correlates of response to immune checkpoint blockade in microsatellite-stable solid tumors. *Nat Genet* **50**, 1271-1281, doi:10.1038/s41588-018-0200-2 (2018).

3 Samstein, R. M. *et al.* Tumor mutational load predicts survival after immunotherapy across multiple cancer types. *Nat Genet* **51**, 202-206, doi:10.1038/s41588-018-0312-8 (2019).

4 Van Allen, E. M. *et al.* Genomic correlates of response to CTLA-4 blockade in metastatic melanoma. *Science* **350**, 207-211, doi:10.1126/science.aad0095 (2015).

5 Wang, F. *et al.* Evaluation of POLE and POLD1 Mutations as Biomarkers for Immunotherapy Outcomes Across Multiple Cancer Types. *JAMA Oncol* **5**, 1504-1506, doi:10.1001/jamaoncol.2019.2963 (2019).

6 Rizvi, H. *et al.* Molecular Determinants of Response to Anti-Programmed Cell Death (PD)-1 and Anti-Programmed Death-Ligand 1 (PD-L1) Blockade in Patients With Non-Small-Cell Lung Cancer Profiled With Targeted Next-Generation Sequencing. *J Clin Oncol* **36**, 633-641, doi:10.1200/JCO.2017.75.3384 (2018).

7 Newman, A. M. *et al.* Robust enumeration of cell subsets from tissue expression profiles. *Nat Methods* **12**, 453-457, doi:10.1038/nmeth.3337 (2015).

8 Thorsson, V. *et al.* The Immune Landscape of Cancer. *Immunity* **48**, 812-830 e814, doi:10.1016/j.immuni.2018.03.023 (2018).

9 Saltz, J. *et al.* Spatial Organization and Molecular Correlation of Tumor-Infiltrating Lymphocytes Using Deep Learning on Pathology Images. *Cell Rep* **23**, 181-193 e187, doi:10.1016/j.celrep.2018.03.086 (2018).

10 Danaher, P. *et al.* Gene expression markers of Tumor Infiltrating Leukocytes. *J Immunother Cancer* **5**, 18, doi:10.1186/s40425-017-0215-8 (2017).

11 Rooney, M. S., Shukla, S. A., Wu, C. J., Getz, G. & Hacohen, N. Molecular and genetic properties of tumors associated with local immune cytolytic activity. *Cell* **160**, 48-61, doi:10.1016/j.cell.2014.12.033 (2015).

12 He, Y., Jiang, Z., Chen, C. & Wang, X. Classification of triple-negative breast cancers based on Immunogenomic profiling. *J Exp Clin Cancer Res* **37**, 327, doi:10.1186/s13046-018-1002-1 (2018).

13 Hanzelmann, S., Castelo, R. & Guinney, J. GSVA: gene set variation analysis for microarray and RNA-seq data. *BMC Bioinformatics* **14**, 7, doi:10.1186/1471-2105-14-7 (2013).

14 Sanchez-Vega, F. *et al.* Oncogenic Signaling Pathways in The Cancer Genome Atlas. *Cell* **173**, 321-337 e310, doi:10.1016/j.cell.2018.03.035 (2018).
