# Supplementary material for: Mutation status of the KMT2 family associated with immune checkpoint inhibitors (ICIs) therapy and implicating diverse tumor microenvironments
Source: Mol Cancer. 2024 Jan 15;23:15. doi: 10.1186/s12943-023-01930-8 (PMC10789049; doi:10.1186/s12943-023-01930-8)
Supplement: Supplementary file 4 — Supplementary Material 4 [file 12943_2023_1930_MOESM4_ESM.docx]

**Supplementary figure**

**
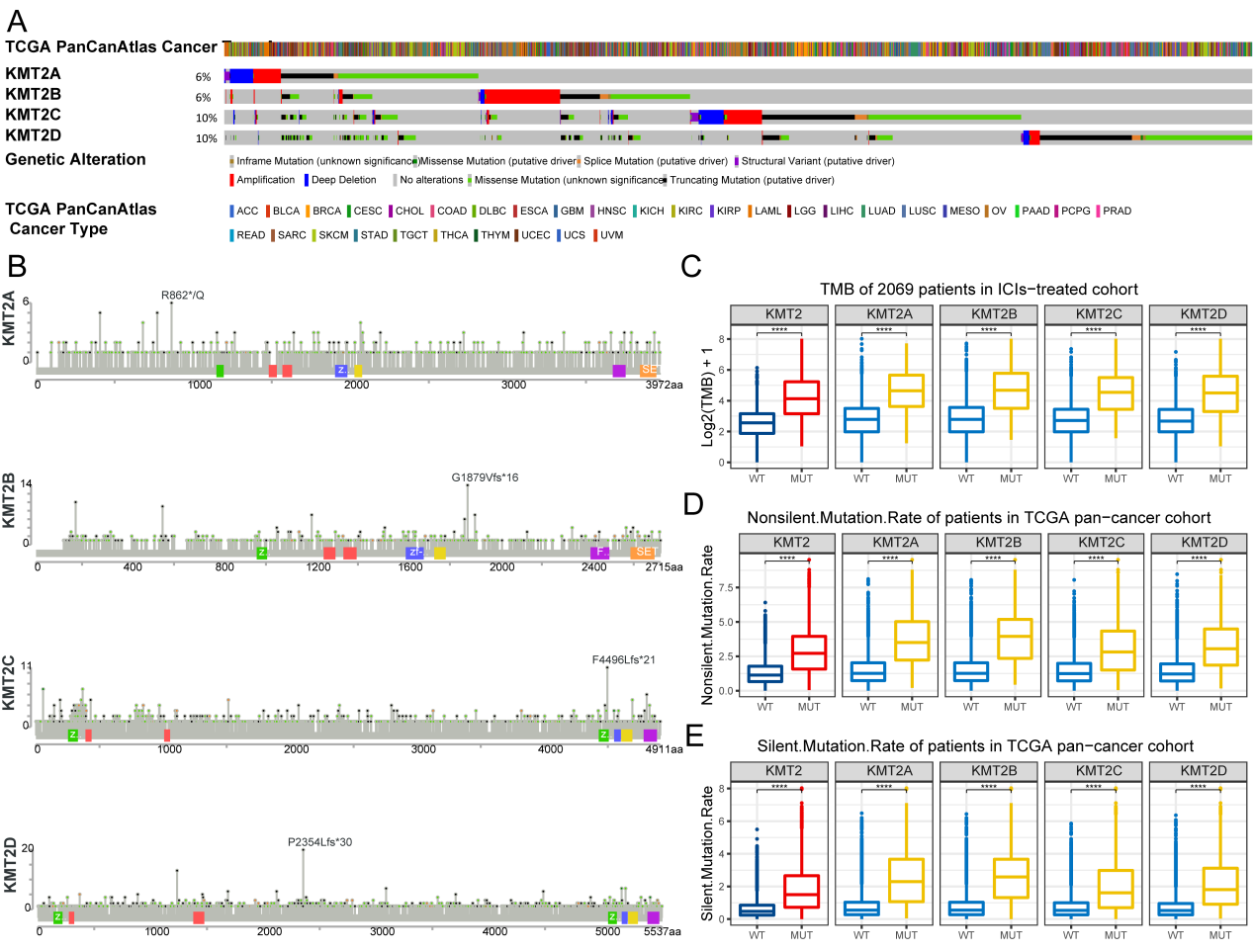
**

**Figure S1**

**A** Oncoprint plot of four KMT2 family members in TCGA pan-cancer cohort. **B** The lollipop plot shows the distribution of mutation sites of the KMT2 family in TCGA pan-cancer cohort. **C** The TMB levels for patients with or without KMT2 family mutations in ICIs-treated cohort(n=2069). **D,E** Non-silent mutation rate and silent mutation rate for patients with or without KMT2 family mutations in TCGA pan-cancer cohort.

**
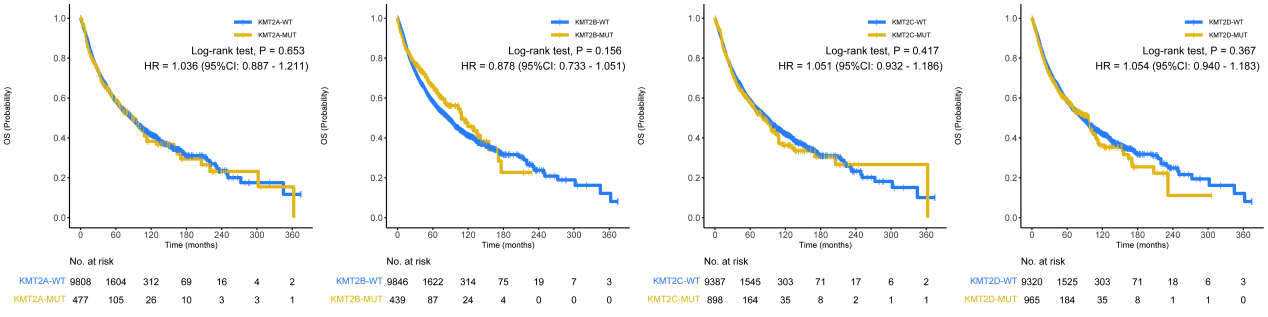
**

**Figure S2**

1. M curves show the difference of OS between patients with or without the four members of KMT2 family mutations in TCGA pan-cancer cohort.


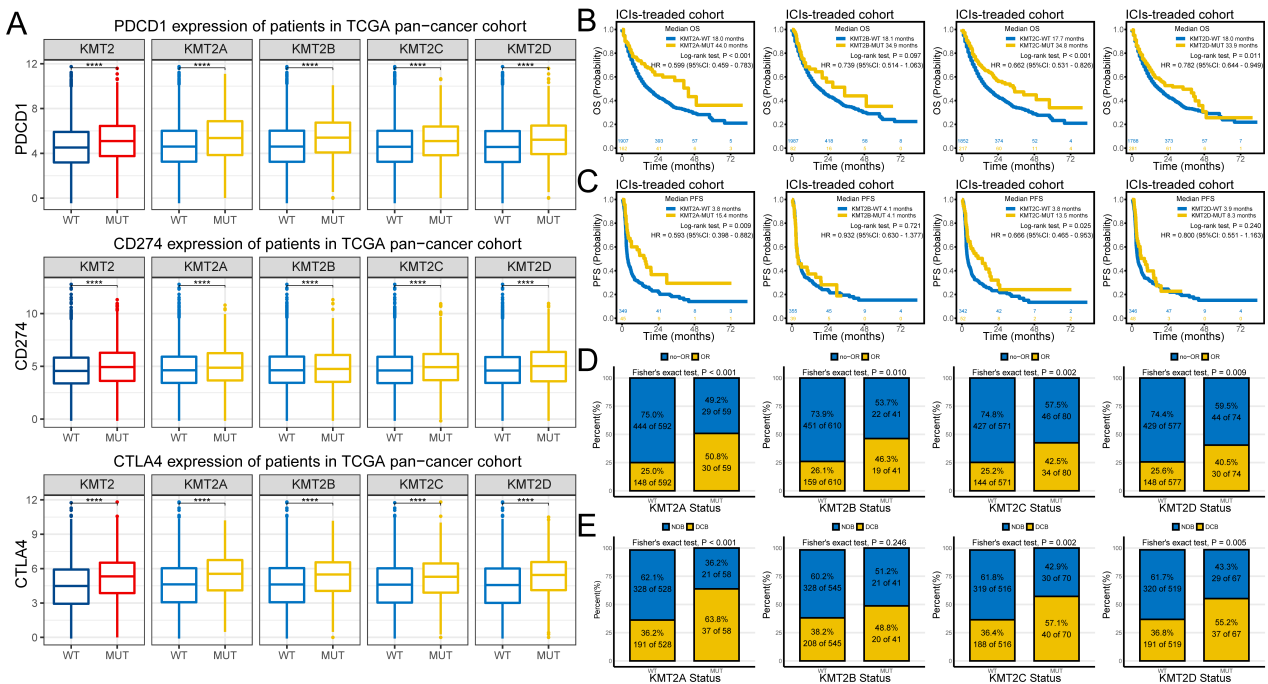


**Figure S3**

**A** The mRNA expression levels of three immune checkpoints(PDCD1, CD274, CTLA4)for patients with or without KMT2 family mutations TCGA pan-cancer cohort. **B,C** K-M curves show the difference of OS and PFS between patients with or without KMT2 family mutations in ICIs-treated cohort(n=2069). **D,E** Patients with KMT2 family mutations received large ORR and DCB rate while receiving ICIs therapy.

**
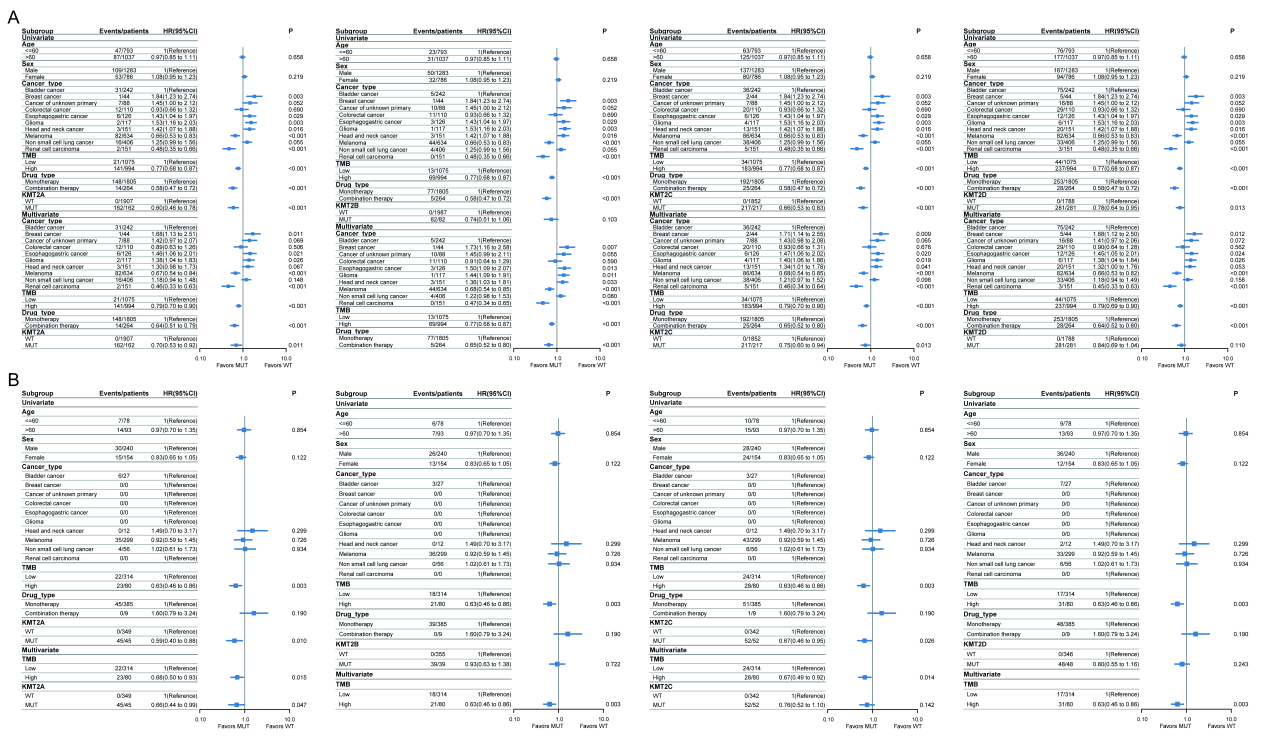
Figure S4**

**A** OS-related univariate and multivariate Cox regression analyses for mutations of KMT2 family members. **B** PFS-related univariate and multivariate Cox regression analyses for mutations of KMT2 family members.


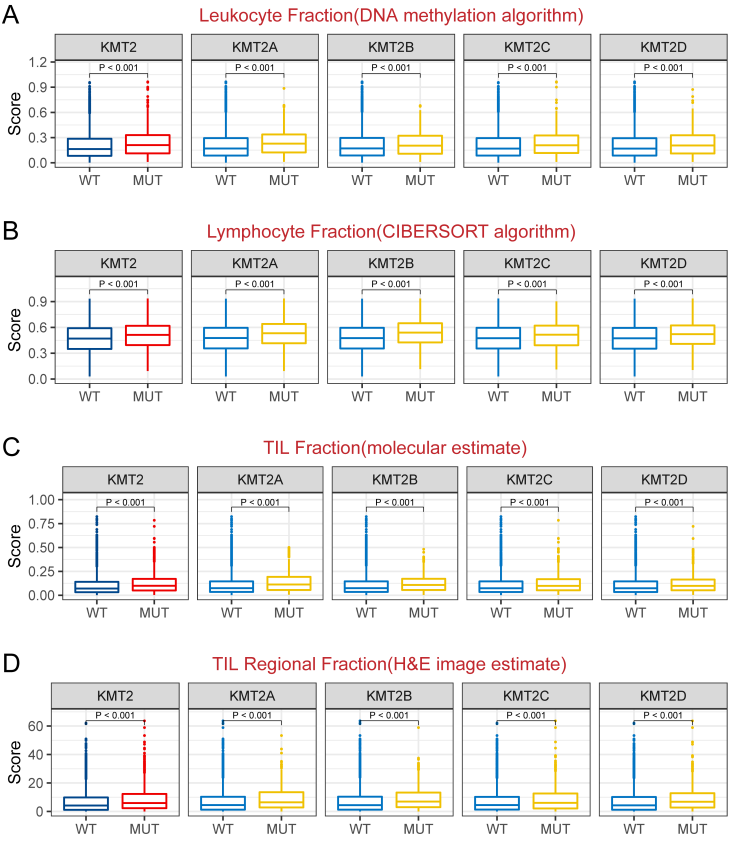


**Figure S5**

Differences of leukocyte fraction(DNA methylation algorithm), lymphocytes fraction(CIBERSORT algorithm), TIL fraction(molecular estimate) and the TIL regional fraction(H&E image estimate) between KMT2-MUT and KMT2-WT tumor.


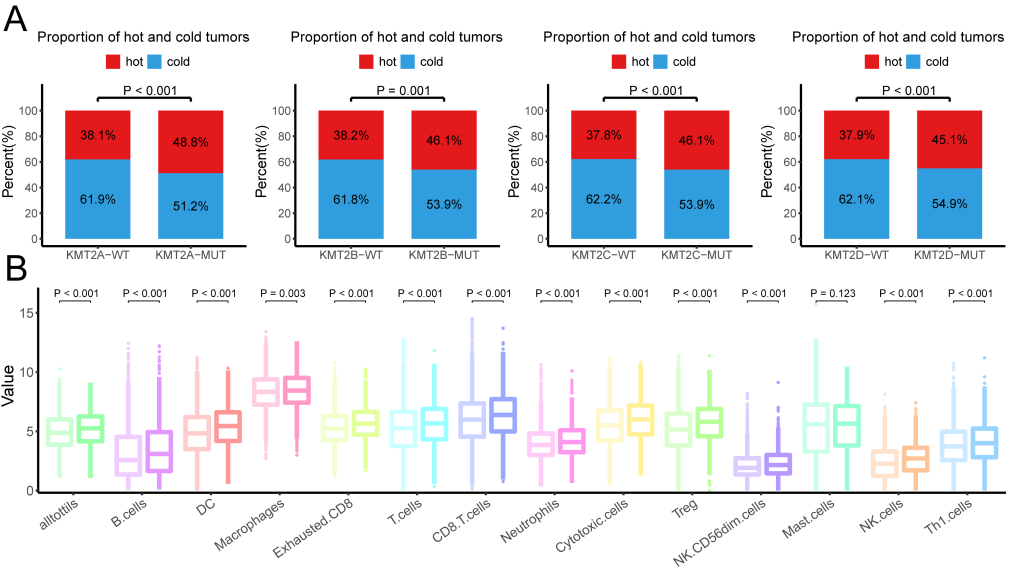


**Figure S6**

**A** Higher proportion of “Hot tumor” were observed in tumor with KMT2 family mutations. **B** Comparison of 14 tumor infiltrating cells scores calculated by Danaher method between KMT2-MUT and KMT2-WT tumor.


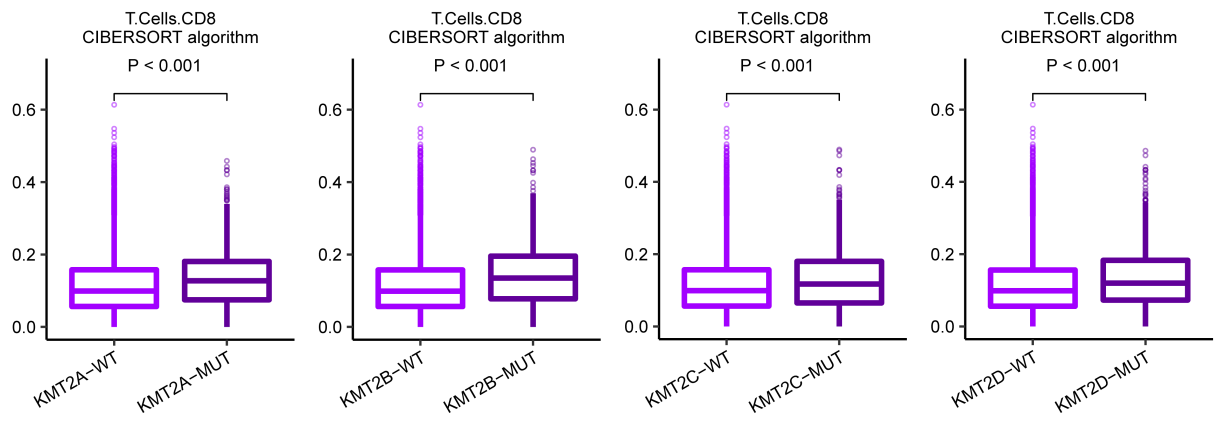


**Figure S7**

Comparison of CD8 T cells fraction(CIBERSORT algorithm) between KMT2(A,B,C,D)-MUT and KMT2(A,B,C,D)-WT tumor.

**Figure S8**

**A** The volcanic diagram provides a more intuitive showcase of higher cell enrichment scores in KMT2-MUT tumor. **B** Correlation heatmap of 29 immune signatures in the KMT2-MUT tumor (top right panel) and KMT2-WT tumor (low left panel). **C** Comparison of correlation coefficient between KTM2A-MUT and KMT2A-WT tumor. **D,E,F** Comparison of CYT, expression levels of PRF1 and GZMA between KMT2-WT and KTM2-MUT tumor. **G** The mRNA expression levels difference of interleukins and receptors, interferons and receptors between KMT2-MUT and KMT2-WT tumor. **H** Ten classical carcinogenic pathways in KMT2-MUT and KMT2-WT tumor.


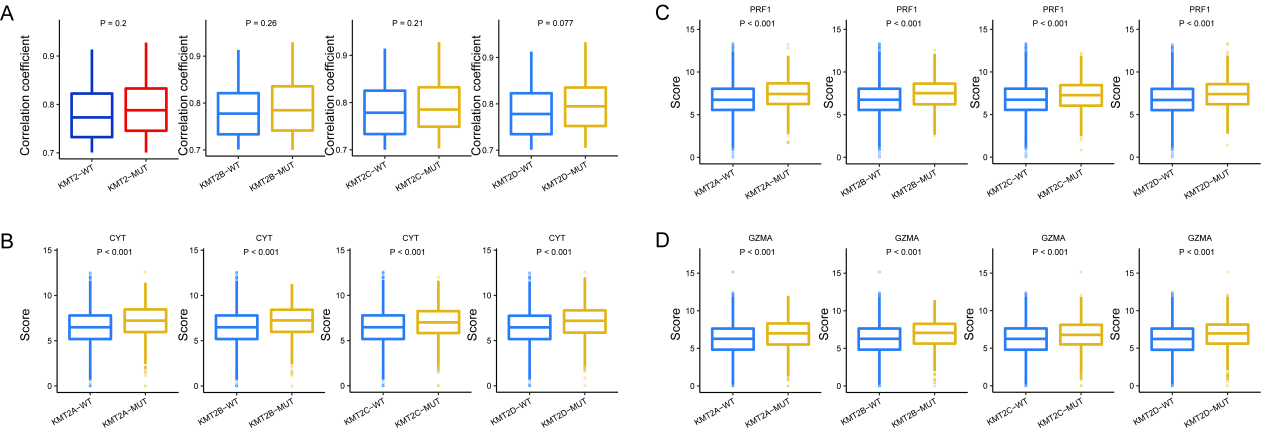


**Figure S9**

**A** Comparison of correlation coefficient between KTM2(B,C,D)-MUT and KMT2(B,C,D)-WT tumor. **C,D,E** Comparison of CYT, expression levels of PRF1 and GZMA between KMT2(A,B,C,D)-WT and KTM2(A,B,C,D)-MUT tumor.
